# Small RNAs in Circulating Exosomes of Cancer Patients: A Minireview

**DOI:** 10.3390/ht6040013

**Published:** 2017-10-06

**Authors:** Stefania Bortoluzzi, Federica Lovisa, Enrico Gaffo, Lara Mussolin

**Affiliations:** 1 Department of Molecular Medicine, University of Padova, 35122 Padova, Italy; enrico.gaffo@unipd.it; 2 Department of Women’s and Children’s Health, University of Padova, 35128 Padova, Italy; federica.lovisa@unipd.it (F.L.); lara.mussolin@unipd.it (L.M.); 3 Istituto di Ricerca Pediatrica Città della Speranza, 35127 Padova, Italy

**Keywords:** exosomes, cancer, small RNA, miRNA, RNA-seq

## Abstract

Extracellular vesicles (EVs) secreted from many cell types play important roles in intercellular communication, both as paracrine and endocrine factors, as they can circulate in biological fluids, including plasma. Amid EVs, exosomes are actively secreted vesicles that contain proteins, lipids, soluble factors, and nucleic acids, including microRNAs (miRNAs) and other classes of small RNAs (sRNA). miRNAs are prominent post-transcriptional regulators of gene expression and epigenetic silencers of transcription. We concisely review the roles of miRNAs in cell-fate determination and development and their regulatory activity on almost all the processes and pathways controlling tumor formation and progression. Next, we consider the evidence linking exosomes to tumor progression, particularly to the setting-up of permissive pre-metastatic niches. The study of exosomes in patients with different survival and therapy response can inform on the possible correlations between exosomal cargo and disease features. Moreover, the exploration of circulating exosomes as possible sources of non-invasive biomarkers could give new implements for anti-cancer therapy and metastasis prevention. Since the characterization of sRNAs in exosomes of cancer patients sparks opportunities to better understand their roles in cancer, we briefly present current experimental and computational protocols for sRNAs analysis in circulating exosomes by RNA-seq.

## 1. Small Non-Coding RNAs

Non-coding small RNAs (sRNAs) are short (~20–30 nt) molecules that can associate with RNAses of the Argonaute family (AGO) to modulate gene expression by targeting complementary transcripts. The repertoire of sRNAs includes endogenous small interfering RNA (endo-siRNA [1]) produced from hybrids between sense and natural antisense transcripts, Piwi-interacting RNA (piRNA [2]) involved in retrotransposon silencing in germ cells but possibly relevant also in cancer cells [3], and the better characterized microRNA (miRNA) and miRNA-like sRNAs.

miRNAs are ~22 nt long important mediators of the posttranscriptional regulation that negatively regulate mRNA stability (by AGO-dependent cleavage of target mRNA and/or destabilization through poly(A) tail shortening) and/or translation via polysomal protein interactions [4]. Moreover, miRNAs exert transcriptional silencing through chromatin modifications [5,6,7]. The regulatory effect of miRNAs can be pervasive since one single miRNA can target transcripts of hundreds different genes, and a single transcript can be regulated by different miRNAs. Target recognition depends on partial sequence complementarity, and specific pairing patterns have been described [8].

In the canonical pathway [9], primary (pri)miRNA transcript nuclear cleavage produces hairpin folded miRNA precursors (pre-miRNAs), whose cytosolic processing generates a miRNA duplex from which one strand (mature miRNA) is incorporated in the RNA-induced silencing complex (RISC) to guide it on targets. Currently, 2588 mature miRNAs from 1881 hairpin precursors are known (MiRbase v.21) [10]. Post processing nucleotide addition, removal, or editing was observed in most miRNA sequences, generating miRNA isoforms (isomiRs [7,11,12]) whose biological significance is emerging [13]. Moreover, alternative processing of miRNA precursors can generate miRNA-offset RNAs (moRNAs) [7,11,12,14]. miRNAs can be also generated through non-canonical mechanisms [15,16], as pre-miRNAs can derive from spliced intronic sequences (miRtrons) [17], or from the processing of housekeeping non-coding RNAs (ncRNAs), such as small nucleolar RNAs (snoRNAs) [18,19], transfer RNAs (tRNAs) [20,21], vault RNAs [22,23], and Y-RNAs [24].

## 2. MicroRNA Functions and Roles in Cancer Development

miRNAs are involved in most physiological processes, playing important roles in cell-fate determination and development [25,26,27]. miRNA expression is regulated at the epigenetic level by transcription factors and by modulation of their biogenesis [28]. Besides, long coding and non-coding RNAs and circular RNA (circRNA) [29,30] competition for miRNA binding regulates miRNA activity on their targets in the so-called competing endogenous RNA (ceRNA) networks [29].

miRNA deregulation through epigenetic modifications, mutations, amplification/deletion, or alteration of the biogenesis machinery has been associated to many pathological processes including tumor formation and progression [31,32]. More than two decades of intensive research showed the involvement of miRNAs in all the processes of cancer cell transformation, with miRNAs acting as oncomirs (e.g., miR-21 has oncogenic properties in many cancer types), tumor suppressors (e.g., the let-7 miRNA family, negatively regulated by NF-κB, represses cell growth), or with dual roles (e.g., miR-182) [31]. The interplay of miRNAs and transcription factors (TF) in mixed regulatory networks [33,34,35,36] and the connections of miRNAs with cancer signaling pathways have been described in last years [33,34,35,36].

Expression profiling identified miRNA signatures associated with specific cancer developmental steps, from normal to metastatic stage and with the acquisition of cancer-specific features [37,38,39]. miRNAs have been linked to senescence bypass of premalignant cells. For instance, the miR-17-92 cluster of oncogenic miRNAs is frequently overexpressed in human cancers and was shown to promote cell proliferation and apoptosis escape and to induce oncogenic transformation, while miR-34a epigenetic silencing facilitates senescence evasion. Tumor angiogenesis, required for malignancy, is under control of miRNAs: miR-221/222 are anti-angiogenic (through the stem cell factor (SCF) and KIT Proto-Oncogene Receptor Tyrosine Kinase (c-Kit) axis), while miR-27b, miR-130a, and miR-126, the last also termed angiomiR, are pro-angiogenic. Tumor progression has been linked to specific miRNA-involving circuits. The most prominent examples are the p53-miRNA and the NF-κB-miRNA regulatory networks (see [31] and references therein). The former controls multiple processes, including cell cycle progression, survival, metabolism, epithelial-mesenchimal transition (EMT), stemness, and angiogenesis. The latter is a critical regulator of pro-inflammatory and stress-like responses that modulates the genetic programs sustaining cell growth, cancer cell survival and cell motility, and regulating EMT and extracellular matrix homeostasis.

Furthermore, many studies connected the expression of specific miRNAs to the activity of the immune suppressive network, of which myeloid-derived suppressor cells are key players. For instance, miR-142-3p downregulation promotes macrophage differentiation and acquisition of immunosuppressive function in tumors [40], and hypoxia-induced miR-210 expression potentiates the tumor-promoting effects of myeloid-derived suppressor cells [41], while both miR-17-5p and miR-20a, by blocking STAT3 expression, alleviate the suppressive potential of myeloid-derived suppressor cells [42].

Metastasis, a process of major clinical relevance as it accounts for most cancer-associated deaths, is supported by the interplay of cancer cells themselves, the tumor stroma, and its microenvironment. In this regard, specific miRNAs, including miR-200 family and miR-205, have been linked to EMT in different cancer types, since they target the transcripts of the EMT inducing TFs Zeb1/2 [43]. As revised in [31], hampered miRNA-processing activity in hypoxia decreases miR-210 expression, which normally represses the initiation of tumor growth, regulating cancer cells adaptation to hypoxic conditions. Another example of a miRNA with pro-metastatic properties is miR-10b, which is under control of Twist1 TF and is associated with mesenchymal features and invasive properties in breast cancer. Several miRNAs are involved in the generation and maintenance of cancer stem cells, as the equilibrium of TF-miRNA regulatory networks controls the acquisition by cancer cells of stem cell-like characteristics associated to the propensity of invading the surrounding tissue and of displaying resistance to therapy. MiR-93 has been shown to inhibit or promote the stem cell phenotype in different cancer types [31].

miRNAs are useful as diagnostic, prognostic, and post-surgery follow up markers [44]. Furthermore, strong research efforts have been focused on miRNA therapeutics against specific features of cancer cells, first of all chemoresistance. Current strategies at different stages of preclinical and clinical development are inhibitory miRNA targeting (by antisense oligonucleotides, miRNA sponges, or small-molecule) and, conversely, restoration of the expression and function of specific miRNAs by synthetic miRNA mimetics [45].

## 3. Extracellular Vesicles Contain, Transport and Deliver small RNAs

Over the past decade, there has been fast growth in studies of secreted membrane vesicles, collectively called extracellular vesicles (EVs), including exosomes, ectosomes, microvesicles, microparticles, apoptotic bodies, and other EV subsets. However, two main populations of EV are shed by living cells: the exosomes (diameter 30–100 nm), formed by inward budding of endosomal membranes and released upon the fusion of multivesicular bodies with the plasma membrane of target cells, and the microvesicles (MV, diameter 100–1000 nm), which bud off directly from the cell membrane [46].

Exosomes have been isolated from diverse body fluids, including semen [47], blood [48], urine [49], saliva [50], breast milk [51], amniotic fluid [52], ascites fluid [53], and cerebrospinal fluid [54]. During the last few years, the importance of exosomes and EVs [55] in intercellular communication has emerged: they serve as vehicles to transfer membrane components, cytosolic proteins, lipids, and RNA between cells. Given the fact that exosomes carry complex biological information, they are implicated in a variety of physiological and pathological conditions. Physiologically, exosomes function as paracrine and endocrine signaling in cell-cell communications.

Physiologically, exosomes function as paracrine and endocrine signaling in cell-cell communications. Exosomes can in fact mediate epithelium-mesenchyme crosstalk in organ development [56,57,58] and are linked to elicitation of specific immune responses, as mast cell-dependent B and T lymphocyte activation is mediated by the secretion of immunologically active exosomes [59].

**Figure 1:**
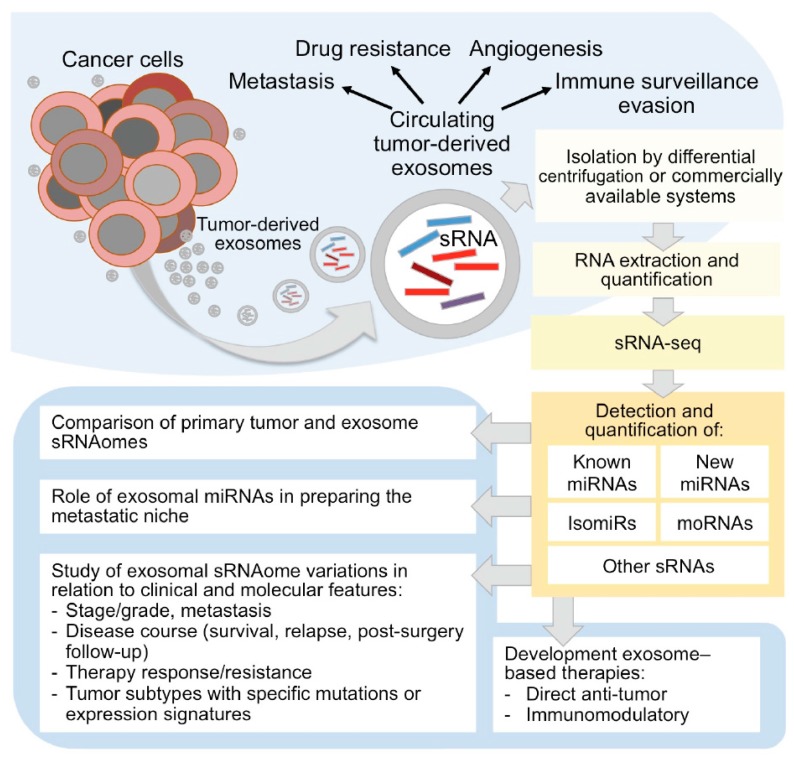
Circulating tumor-derived exosomes sRNAome characterization by sRNA-seq in cancer research. As sketched in the upper panel, tumor-derived exosomes carry a complex cargo, including sRNAs, and recent studies are disclosing the intriguing roles of exosomes as active players in tumor progression and spreading, stimulating tumor cell growth, suppressing the immune system response, inducing angiogenesis and metastatic process, and as emerging tumor biomarkers. The study of sRNAome carried by tumor-derived circulating exosomes by sRNA-seq (right part of the figure) can inform on micro (mi)RNAs, isomiRs and other sRNA species, including miRNA-offset (mo)RNAs, and allow to understand the role of exosomes in cancer development and metastasis, in therapy resistance, and to explore their usefulness as markers, therapeutic targets or agents.

Recently, studies based on deep sequencing technology (RNA-seq) revealed the presence into exosomes of a broad spectrum of sRNA types [60], in particular miRNAs (Figure 1). Exosomes could exhibit different sRNA profiles compared to the producing cells, suggesting a selective incorporation of specific sRNA with definite regulatory functions into these EVs [61]. Small RNA molecules can be even transferred by extracellular vesicles between organisms in inter-species crosstalk, as in the host-microbiota interactions in the gut [56].

## 4. Exosomes Roles in Cancer Development and Progression and Relevance as Biomarkers

The first study demonstrating that tumor cells release a large amount of exosomes dates back to 1983 [62] were first thought to participate in the selective removal of cellular trash. However, the interest in these EVs exploded in the last decade, and molecular and functional characterization of exosomes-associated miRNAs has been described in different solid cancers, such as breast cancer, melanoma, and lung cancer [2,63,64]. To date, several studies showed the intriguing role of exosomes as active players in tumor progression and spreading, stimulating tumor cell growth, suppressing the immune system response, inducing angiogenesis and metastatic process, and considered them as emerging tumor biomarkers [65,66] (Figure 1).

In non-small cell lung cancer, exosomal miR-21 and miR-188 overexpression correlated with a negative prognosis, defining a combined signature possibly representing a new independent prognostic biomarker for relapse-free survival and overall survival [67]. Exosomal miRNA profiling has proved useful to distinguish pancreatic carcinomas from benign pancreatic tumors and chronic pancreatitis [68], whereas expression of miR-34a was associated to drug resistance in prostate cancer [69]. It has been shown that pharmacologic inhibition of BRAFV600 in malignant melanoma cell alters the microRNA cargo in the vesicular secretome, with an increase of miR-211-5p, linked to the activation of survival pathway [70]. The usefulness of plasma vesicle miRNAs for therapy response and relapse monitoring in Hodgkin lymphoma patients was recently reported [71]. In the future, the use of exosomes as biomarkers is expected to prove advantageous and reduce the use of invasive surgery for both diagnosis and monitoring of disease course in cancer patients.

The biology of cancer closely depends on the surrounding tumor microenvironment. The importance of intercellular communication in the regulation, induction, and maintenance of cancer cells, and in the promotion of invasion has been the subject of several studies. In this regard, the role of exosomal miRNAs within the tumor microenvironment is emerging to be of paramount relevance (Figure 1). Recently, Challagundla et al. demonstrated that the frequently observed increase of miR-155 in solid tumors may be due to the presence of pro-tumorigenic immune cells in the tumor stroma rather than to the expression of the miRNA by tumor cells themselves [72]. The authors also demonstrated the unique role of exosomal miR-21 and miR-155 in the crosstalk between neuroblastoma cells and human monocytes and their association to chemotherapy resistance through a novel exosomal miR-21/TLR8-NF-кB/exosomal miR-155/TERF1 signaling pathway [72]. Furthermore, tumor exosomes have recently been shown to target non-transformed cells in pre-metastatic organs and modulate their phenotype predominantly through transferred miRNAs [73]. Sanchez and co-authors demonstrated that miR-100 and miR-21 were the most abundant exosomal miRNAs in human prostate cancer. The authors proved that miR-100/miR-21 transfection in normal prostate fibroblasts increased the expression of metalloproteinases-2/9/13 and RANKL and fibroblast migration, contributing to pre-metastatic niche preparation, suggesting a potential role of exosomal miRNAs as a therapeutic target [74]. Hypoxic oral squamous cell carcinoma produces miR-21-rich exosomes that deliver to normoxic cells eliciting a premetastatic phenotype [75].

## 5. Development of Exosome-Based Therapies

Since exosomes represent natural vectors for bio-molecular transfer, there is growing interest in using them as therapeutic delivery vehicles. In 2011, Mittelbrunn et al. stably over-expressed miR-335 in J77 T-cells, which do not express miR-335, and demonstrated that the T-cell derived exosomes contained and could transfer functional miR-355 to recipient cells that did not express miR-355 [76]. These experiments demonstrated that cultured cells could be induced to package miRNAs into exosomes. Exosomes can be tumor-suppressive when loaded with anti-tumor miRNAs, suggesting that they might be used for therapeutic purposes.

Shimbo et al. employed mesenchymal stem cells to package into exosomes miR-143, a tumor-suppressor miRNA characteristically down-regulated in the 143B human osteosarcoma cell line. In this study, exosomal miR-143 was easily delivered to recipient cells and proved to suppress migration of 143B osteosarcoma cells [77]. Munoz et al. reported that mesenchymal stem cells transfected with an anti-miR-9 in non-contact co-culture with glioblastoma cells transferred the anti-miR to cancer cells via secreted exosomes [78]. These exosomes conferred temozolomide chemosensitivity modulated by miR-9, suggesting that exosomes might change the response to chemotherapeutics of target cells [78].

The current research efforts regarding development of effective and safe EV-based therapies are witnessed by recent interesting publications, including a position paper of the International Society for Extracellular Vesicles on issues and potentialities of the application of EV-based therapeutics in clinical trials [79], a review discussing the current state-of-the-art and future developments of best-practices for EV-based therapy, also citing direct and immunomodulatory anti-tumor strategies [80], and a review focused on EVs in hematological malignancies [81].

## 6. Experimental and Computational Procedures to Study Circulating Exosomal small RNAs by RNA-Seq

Small RNA profiling in circulating exosomes by RNA-seq holds high potential for miRNA detection, discovery and characterization. The quantification of miRNAs, other sRNAs, and isomiRs in exosomes and in cancer tissues can be very useful to understand the roles of tumor derived extracellular vesicles and to appreciate the mechanisms of their derivation from tumor cells and of cargo selection. The study of exosomes in patients with different survival, tumor aggressiveness, therapy response, or resistance can inform on the possible correlations between exosomal cargo and disease features (Figure 1).

### 6.1. Exosome RNA Isolation and Sequencing

To date, most published studies of EV from biofluids or cell culture have employed differential centrifugation with or without size filtration to concentrate and partially purify EVs [82]. This method comprises a series of high-speed spins (~100,000× *g*) to selectively sediment exosomes and/or EVs from solution. Recently, several commercial kits such as ExoQuick (System Bioscience, Palo Alto, CA, USA) and Exo-Spin (Cell Guidance, Cambridge, UK) were developed to facilitate sedimentation of exosomes during low speed centrifugation by inducing precipitation of vesicles with polyethylene glycol or similar substances [83,84]. Another system, the exoRNeasy Serum/Plasma kit (Qiagen, Hilden, Germany), uses membrane affinity spin columns to efficiently isolate exosomes and other EVs from pre-filtered biological fluids [85].

According to the Qiagen protocol, isolation of exosomes can be performed starting from 1 mL of plasma and vesicles eluted in 300 µL of Buffer XE. It is advisable to preserve at least 100 µL of vesicle suspension for exosome validation and quantification by Tunable Resistive Pulse Sensing system (qNano, (Izon Science, Oxford, UK)) or Nanoparticle Tracking Analysis (Nanosight, Malvern Instruments, Malvern, UK). Conventional Western blotting may also be performed to confirm the presence of proteins enriched in EVs, such as tetraspanins CD9, CD63 and CD81. Exosome morphology can be assessed by electron microscopy or by atomic force microscopy [82]. The remaining 200 µL of vesicles can be processed for RNA extraction by adding 5 volumes of Qiazol reagent to each sample and by loading the mixtures into a new exoEasy spin column.

It should be noted that exosomal RNA yield is very low and in some case the concentration can be below the spectrophotometer and fluorimeter sensitivities. In light of this, it is mandatory to assess RNA concentration and integrity using a high sensitive method, for instance the Agilent RNA Picochip (Agilent Technologies, Santa Clara, CA, USA). The resulting RNA size ranges are typically below 200 bp, with no ribosomal species detectable [79].

Sequencing libraries specific for sRNAs can be prepared starting from 2–10 ng of RNA, by using the NEBNext Multiplex Small RNA library Prep Set for Illumina (New England Biolabs, Ipswich, MA, USA), with slight modifications for low input RNA, as previously published [86]. The suggested depth for sequencing on an Illumina platform is around 10–15 millions of raw reads for reaching the plateau of unique miRNA detectable [86]. However, if a sufficient amount of RNA is available, a higher sequencing depth could better support the detection of isomiRs and rare sRNA species.

### 6.2. Bioinformatic Analysis of Exosomal sRNA-Seq Data

As for traditional high-throughput sequencing of sRNAs (sRNA-seq), exosomal sRNA-seq data analysis may serve different purposes depending on the study design. A first achievement is the identification and expression quantification of the known miRNAs loaded into exosomes. Given that human miRNAs are largely annotated, restricting the analysis to known elements may nevertheless consider most miRNAs of the selected cargo and the procedure can be as simple as mapping the reads to the reference genome, followed by intersecting the alignments with the annotation and counting.

In addition to providing ready to use workflows, tools devoted to miRNA characterization from miRNA-seq experiments usually improve accuracy on their predictions and estimates by performing quality checks and applying appropriate stringency filters. Despite the different strategies implemented, many tools consider and share some key steps of the analysis process. The raw reads are screened for the presence of adapter fragments, which spot the reads bearing sRNA sequences. Non-adapter reads are discarded and for the remaining ones the adapter nucleotides are trimmed with tools like FASTX-Toolkit (http://hannonlab.cshl.edu/fastx_toolkit/index.html), Trimmomatic [87] or Flexbar [88]. Trimmed reads of 15–30 nt are then selected to enrich possible miRNA sequences, yielding a clean read set.

After raw reads preprocessing, the clean reads are mapped to the genome with short read aligners like Bowtie [89] or BWA [90], tolerating at most two mismatches to account for SNPs or edited bases. Expression of known miRNAs is quantified by counting the alignments intersecting the annotation. For the prediction of the novel miRNA species, many methods compute the probability that the genomic region surrounding a block of alignments folds into a miRNA hairpin-like structure. In addition, filtering and reporting of the predictions is specific of each software tool.

More sophisticated methods allow a deeper characterization of the miRNome by predicting novel precursors and sRNA species, such as moRNAs [12,91], or considering the inspection of miRNA sequence isoforms [92,93]. IsomiRs can possibly discriminate cancer types [94,95] but require attention in the study design [96]. The presence of specific isomiRs, due to 3′ end posttranscriptional modifications, in exosomes compared to cells has been described [97].

However, miRNA can constitute only a fraction of sRNAs carried by exosomes. Indeed, a recent study of sRNAs in exosomes of on EBV-driven LCLs and B cell lymphoma cell lines, beyond identifying specific miRNAs enriched in exosomes fractions, showed that miRNAs represent around 50% of the sRNA pool in cellular samples and account for 20% in exosomes, in which other sRNAs are enriched, including species derived from tRNAs, rRNAs, Y RNAs, and vault RNAs with different class distributions in different cell types [97]. Clearly, the non-miRNA fraction of exosomal sRNA deserves further investigation. Several bioinformatics tools can report the presence of sRNA produced by regulated processing or by degradation of other RNAs [98] that, given the broad range of sRNAs contained in EV [97,99] and the risk of contaminants [100], may be relevant in exosome sRNA characterization.
